# Supplementary material for: Assessment of polygenic architecture and risk prediction based on common variants across fourteen cancers
Source: Nat Commun. 2020 Jul 3;11:3353. doi: 10.1038/s41467-020-16483-3 (PMC7335068; doi:10.1038/s41467-020-16483-3)
Supplement: Supplementary file 3 — Reporting Summary [file 41467_2020_16483_MOESM3_ESM.pdf]

nature research

Corresponding author(s): Nilanjan Chatterjee

Last updated by author(s): Mar 10, 2020

Reporting Summary

Nature Research wishes to improve the reproducibility of the work that we publish. This form provides structure for consistency and transparency in reporting. For further information on Nature Research policies, see Authors & Referees and the Editorial Policy Checklist .

Statistics

For all statistical analyses, confirm that the following items are present in the figure legend, table legend, main text, or Methods section.

n/a	Confirmed	
☐	☒	The exact sample size (*n*) for each experimental group/condition, given as a discrete number and unit of measurement
☒	☐	A statement on whether measurements were taken from distinct samples or whether the same sample was measured repeatedly
☒	☐	The statistical test(s) used AND whether they are one- or two-sided<br>*Only common tests should be described solely by name; describe more complex techniques in the Methods section.*
☒	☐	A description of all covariates tested
☒	☐	A description of any assumptions or corrections, such as tests of normality and adjustment for multiple comparisons
☐	☒	A full description of the statistical parameters including central tendency (e.g. means) or other basic estimates (e.g. regression coefficient) AND variation (e.g. standard deviation) or associated estimates of uncertainty (e.g. confidence intervals)
☒	☐	For null hypothesis testing, the test statistic (e.g. *F*, *t*, *r*) with confidence intervals, effect sizes, degrees of freedom and *P* value noted<br>*Give P values as exact values whenever suitable.*
☒	☐	For Bayesian analysis, information on the choice of priors and Markov chain Monte Carlo settings
☒	☐	For hierarchical and complex designs, identification of the appropriate level for tests and full reporting of outcomes
☐	☒	Estimates of effect sizes (e.g. Cohen's *d*, Pearson's *r*), indicating how they were calculated

Our web collection on statistics for biologists contains articles on many of the points above.

Software and code

Policy information about availability of computer code

Data collection	No software was used for data collection. We are using the summary GWAS data.
Data analysis	All analysis coded are deposited in: https://github.com/yandorazhang/CancerEffectSize

For manuscripts utilizing custom algorithms or software that are central to the research but not yet described in published literature, software must be made available to editors/reviewers. We strongly encourage code deposition in a community repository (e.g. GitHub). See the Nature Research guidelines for submitting code & software for further information.

Data

Policy information about availability of data

All manuscripts must include a data availability statement. This statement should provide the following information, where applicable:

- Accession codes, unique identifiers, or web links for publicly available datasets
- A list of figures that have associated raw data
- A description of any restrictions on data availability

The data that support the findings of this study are available by application from the participating consortia: BCAC (bcac@medschl.cam.ac.uk), BEACON (P Gharahkhani), ColonCFR (M Jenkins), GECCO/CORECT (U Peters), ECAC (TA O'Mara), GenoMEL (M Iles), GICC (R Houlston), ILLCO/Integral (C Amos), InterLymph (S Berndt), OCAC (PDP Pharoah), Oral Cancer GWAS (P Brennan), PANC4/PanScan (LT Amundadottir), PRACTICAL (Data Access Committee/http://practical.icr.ac.uk/), Renal Cancer GWAS (MP Purdue, P Brennan), TECAC (KA McGlynn). For breast and prostate cancers, summary GWAS data can also be downloaded from http://bcac.ccge.medschl.cam.ac.uk/bcacdata/oncoarray/gwas-icogs-and-oncoarray-summary-results/ and http://practical.icr.ac.uk/blog/?page_id=8164

Field-specific reporting

Please select the one below that is the best fit for your research. If you are not sure, read the appropriate sections before making your selection.

- [x] Life sciences
- [ ] Behavioural & social sciences
- [ ] Ecological, evolutionary & environmental sciences

For a reference copy of the document with all sections, see nature.com/documents/nr-reporting-summary-flat.pdf

Life sciences study design

All studies must disclose on these points even when the disclosure is negative.

Sample size	We accessed summary-statistics from largest GWAS available to date for each cancer. Number of cases/controls (cancer sites) : 3100/7667 (CLL), 3914/6718 (Esophageal), 3558/13970 (Testicular), 6034/6585 (Oropharyngeal), 8638/12,217 (pancreas), 10,784/20,407 (Renal), 12,488/18,169 (Glioma), 12,874/23,203 (Melanoma), 17,050/19,529 (Colorectal), 12,906/108,979 (Endometrial), 22,406/40,951 (Ovarian), 29,266/56,450 (Lung), 79,148/61,106 (Prostate), 108,067/88,386 (Breast). The sample size of these GWAS study are the largest existing GWAS studies.
Data exclusions	We used pre-determined exclusion criterion that we had used earlier in the paper titled "Estimation of complex effect-size distributions using summary-level statistics from genome-wide association studies across 32 complex traits" (Nature Genetics 2018). Across all cancers, we first restricted analysis to SNPs within a set of reference ~1.07 million SNPs included in the HapMap3 with MAF>0.05 in the 1000 Genome European data. Second, we excluded SNPs having substantial amounts of missing genotype data: sample sizes less than 0.67 times the 90th percentile of the distribution of sample sizes across all SNPs. Third, we excluded SNPs within the major histocompatibility complex (MHC) region (i.e., SNPs between 26,000,000 and 34,000,000 base pairs on chromosome six). The filtering steps are applied to ensure the effect-size distribution estimation are not influenced by outlier SNPs with unsually large effects or/and imprecise estimate of effect due to rare allele frequency of missing genotype data in the original study.
Replication	Not applicable. We are not performing any discovery analysis based on hypothesis testing. The report represents descriptive analysis of effect-size distribution associated with risk of cancers associated with common variants. The concept of replication does not apply.
Randomization	Not applicable. Data comes from observation genome-wide association studies.
Blinding	Not applicable. We are dealing with summary statistics data from observation GWAS.

Reporting for specific materials, systems and methods

We require information from authors about some types of materials, experimental systems and methods used in many studies. Here, indicate whether each material, system or method listed is relevant to your study. If you are not sure if a list item applies to your research, read the appropriate section before selecting a response.

Materials & experimental systems

n/a	Involved in the study	
x		Antibodies
x		Eukaryotic cell lines
x		Palaeontology
x		Animals and other organisms
x		Human research participants
x		Clinical data

Methods

n/a	Involved in the study	
x		ChIP-seq
x		Flow cytometry
x		MRI-based neuroimaging
